# Do End-of-Rotation and End-of-Shift Assessments Inform Clinical Competency Committees’ (CCC) Decisions?

**DOI:** 10.5811/westjem.2017.10.35290

**Published:** 2017-12-13

**Authors:** Linda Regan, Leslie Cope, Rodney Omron, Leah Bright, Jamil D. Bayram

**Affiliations:** * Johns Hopkins University Department of Emergency Medicine, Baltimore, Maryland; † Johns Hopkins University, Department of Oncology, Baltimore, Maryland

## Abstract

**Introduction:**

Clinical Competency Committees (CCC) require reliable, objective data to inform decisions regarding assignment of milestone proficiency levels, which must be reported to the Accreditation Council for Graduate Medical Education. After the development of two new assessment methods, the end-of-shift (EOS) assessment and the end-of-rotation (EOR) assessment, we sought to evaluate their performance. We report data on the concordance between these assessments, as well as how each informs the final proficiency level determined in biannual CCC meetings. We hypothesized that there would be a high concordance level between the two assessment methods, including concordance of both the EOS and EOR with the final proficiency level designation by the CCC.

**Methods:**

The residency program is an urban academic four-year emergency medicine residency with 48 residents. After their shifts in the emergency department (ED), residents handed out EOS assessment forms asking about individual milestones from 15 subcompetencies to supervising physicians, as well as triggered electronic EOR-doctor (EORd) assessments to supervising doctors and EOR-nurse (EORn) to nurses they had worked with after each two-week ED block. EORd assessments contained the full proficiency level scale from 16 subcompetencies, while EORn assessments contained four subcompetencies. Data reports were generated after each six-month assessment period and data was aggregated. We calculated Spearman’s rank order correlations for correlations between assessment types and between assessments and final CCC proficiency levels.

**Results:**

Over 24 months, 5,234 assessments were completed. The strongest correlations with CCC proficiency levels were the EORd for the immediate six-month assessment period prior (r_s_ 0.71–0.84), and the CCC proficiency levels from the previous six-months (r_s_ 0.83–0.92). EOS assessments had weaker correlations (r_s_ 0.49 to 0.62), as did EORn (r_s_ 0.4 to 0.73).

**Conclusion:**

End-of-rotation assessments completed by supervising doctors are most highly correlated with final CCC proficiency level designations, while end-of-shift assessments and end-of-rotation assessments by nurses did not correlate strongly with final CCC proficiency levels, both with overestimation of levels noted. Every level of proficiency the CCC assigned appears to be highly correlated with the designated level in the immediate six-month period, perhaps implying CCC members are biased by previous level assignments.

## INTRODUCTION

In the “Milestone Project” for assessing resident physicians’ competencies,^1^ the determination of milestone proficiency is the responsibility of the Clinical Competency Committees (CCC). To meet this obligation our CCC, composed of our core emergency medicine (EM) educational faculty, meets twice a year. It seeks to rely on objective measures to select one of the five levels of ascending proficiency that best represents each resident’s individual performance during the preceding six months of training.^2^ While suggested assessment methods are provided for each of the subcompetencies within an individual specialty’s milestones,^3^ there are no clear current best practices regarding which assessments are most likely to provide the most useful and valid data to CCCs in the determination of the proper proficiency level.

Previous reports have noted that end-of-shift (EOS) assessments, if used in isolation, yield falsely elevated proficiency levels.^4^ Schott et al. failed to validate the results of direct observation using either a checklist tool or a milestone proficiency-level tool when used in video review of a critical patient encounter with varying levels of trainees. They cited significant issues with both rater error and instrument error.^5^

We developed a multi-modal milestone evaluation program geared at obtaining objective data for CCC usage. In this study we provide a description of the performance of the two predominant assessment methods used in this new milestone evaluation program: (1) the brief EOS assessment collected in paper form at the end of a shift after direct supervision; and (2) the end-of-rotation (EOR) global assessment collected in electronic form. We report data on the concordance between EOS and EOR assessments, as well as how each informs the final proficiency level determined in biannual CCC meetings. We hypothesized that there would be a high concordance level between the two assessment methods, including concordance of both the EOS and EOR with the final proficiency level designation by the CCC.

## METHODS

The study site is an urban academic institution, home to a four-year EM residency with 48 residents and 42 full-time faculty members across two large medical centers. Institutional review board approval was obtained. In short, the EOS assessment involved residents handing out individual assessment sheets comprised of 9–11 individual “milestone” questions, taken from 15 subcompetencies, to supervisory doctors after a shift. These pocket notebooks contained 10 sets of each 8-sheet assessment packet. Assessor identity was not tracked on the EOS. The EOR assessment allowed residents to electronically trigger an online assessment focused on global performance after two weeks of an emergency department (ED) rotation. The EOR for supervisory doctors (EORd) sent the full five levels of ascending proficiency from 16 subcompetencies for supervisory doctors and from four subcompetencies for nurses (EORn). Reports were run for both EOR and EOS assessments after each six-month period to calculate proficiency levels for each of the applicable subcompetencies, and information was provided to members of the CCC.

**Population Health Research Capsule**

What do we already know about this issue?

End-of-shift assessments are thought to provide artificially inflated grades when used to assess trainees, yet residency programs use them to provide information to Clinical Competency Committees (CCC).

What was the research question?

Is there concordance between end-of-shift and end-of-rotation assessments with each other and final proficiency levels assigned by the CCC?

What was the major finding of the study?

End-of-rotation assessments completed by supervising doctors are most highly correlated with final CCC proficiency level designations, while end-of-shift assessments overestimate levels.

How does this improve population health?

Providing valid assessment data to CCCs helps residency programs develop appropriate, targeted development and remediation to trainees, maximizing their patient outcomes.

Similar to a grant review, each CCC member was assigned primary responsibility for up to six residents, reported a summary of the data after review, and suggested proficiency levels to the group. Final proficiency levels were determined after group discussion with guidance from the CCC leader. To determine correlations, aggregate data for the EORd, EORn, EOS, and final CCC proficiency levels were obtained for each of the four six-month time frames. We calculated Spearman’s rank order correlations for correlations between assessment types and between assessments and final CCC proficiency levels. Correlations were considered “very strong” for r_s_ > 0.8, “strong” for r_s_ =0.6–0.79, “moderate for r_s_ = 0.40–0.59, “weak” for r_s_ = 0.20–0.39 and “very weak” for r_s_ < 0.2. We calculated p-values and used the Bonferonni correction to account for the many correlations, with p-values below 0.0005 considered statistically significant.

## RESULTS

A total of 5,234 assessments were completed over 24 months. The EORd accounted for 1,330 assessments, the EORn accounted for 509, and the EOS accounted for 3,395. Table 1 presents the annual completion rates by each assessment type by resident year. Spearman’s rank order correlations between the EOS and EOR assessments are reported in Table 2. Please note that each is aggregated and reported twice a year (December and May) and hence the designation of the month initial and year. For example, EOS.M14 indicates the EOS assessment for May 2014. Furthermore, the EOR assessments were reported separately for physicians and nurses, hence the designation end-of-rotation by doctor (EORd) and end-of-rotation by nurse (EORn).

As demonstrated in Table 2, the EOS and EOR assessments did not have strong correlations, with values ranging from −0.17 to 0.65. Taken within each corresponding timeframe (December or May of the same year), the correlations tended to be better overall. EOS assessments were more strongly correlated with EOR assessments performed by physicians as compared to those performed by nurses. The range of correlations between EOS and EOR performed by nursing was −0.17 to 0.54, while the range of correlations between EOS and EOR performed by physicians was 0.01 to 0.65. Table 3 shows the correlations between the EOR assessments performed by nurses and those performed by physicians.

The final assigned level of proficiency for each subcompetency (designated as CCC.XXX with the same month and year designation as above) is found to be best correlated with EOR assessments performed by physicians for that particular period (Table 4). For example, the CCC assessment for May 2014 (CCC.M14) had a very strong correlation with EOR data from doctors run in May 2015(EORd.M14) (r_s_=0.85). Furthermore, the correlations between EOR assessments performed by physicians and the CCC proficiency level improved temporally up to that particular period. For example, the correlation between CCC.M14 and EORd.D13 was 0.7, and this value improved to 0.85 when correlated with EORd.M14. Similarly, for CCC.M15, the correlations were 0.46, 0.71, 0.81, and 0.84 with EORd.D13, EORd.M14, EORd.D14, and EORd.M15. The correlations of EOS assessments with CCC proficiency levels remain relatively weak, ranging from 0.49 to 0.62. Similarly, the correlations of EOR assessments performed by nurses had modest correlations with CCC proficiency levels, ranging from 0.4 to 0.73.

Looking at the CCC correlations in Table 5 across time, each CCC level of proficiency is very strongly correlated with the assigned CCC proficiency level in the previous time period. For example, the final CCC proficiency level from May of 2015 (CCC.M15) was very highly correlated with the final CCC proficiency level from the previous December in 2014 (CCC.D14) with a value of 0.92. In particular, CCC levels are highly correlated within a given academic year, somewhat less so across academic years, with diminishing association over time.

Across post-graduate year levels (PGY) 1 through 4, we noticed that correlations between the CCC proficiency levels and EORd by physicians were the highest (range 0.74–0.85), compared to CCC proficiency levels correlated with EOS and EORn (Table 5). P-values are less than 0.00001 unless otherwise indicated.

Looking at correlations across various subcompetencies in Table 6, we noted that whenever multiple data sources (EORd, EOS, and EORn) were used to assess an individual subcompetency, the correlation for the CCC proficiency levels across all of these subcompetencies was highest with the EORd compared to the two other data sources. We also noted that the correlations between CCC level of proficiency and EOR assessments by nurses are moderately strong in the four applicable subcompetencies that were chosen with r_s_=0.66, 0.71, 0.65, and 0.57 for multi-tasking, patient-centered communication, team management and professional values (compassion, integrity), respectively.

## DISCUSSION

The development and use of assessment tools for trainee assessment is a critical function of all residency training programs. The development of formal CCCs forced programs to re-evaluate their assessment methods and to determine whether the information being collected was both reliable and valid for use in the determination of proficiency levels for residents, at each stage of training.

### Predictors of Final Recommended CCC Proficiency Level by Assessment Type

While many EM residency programs, including ours, use the EOS assessments that are publicly available via the Council of Residency Emergency Medicine Residency Directors (CORD-EM) website,^6^ the literature calls into question the use of this type of assessment. Warrington et al. (the original developers of the forms available on the CORD-EM site) published results noting only slight to fair inter-rater agreement in a video- based study in which educators at a national conference scored a “resident encounter” using the EOS form.^7^ Another study of EOS assessments, although completed electronically, is described by Dehon et al. in the literature and reports that their EOS assessments in EM yielded inflated proficiency levels when used in isolation and when compared to the final CCC recommended proficiency level.^4^ Our findings corroborate this notion, as we found that EOS assessments were not strongly correlated with final CCC proficiency levels, yielding significantly inflated proficiency levels when compared to the final rankings.

In our study, what mattered most for the final recommended proficiency level by the CCC was the EOR assessment performed by doctors (EORd) for that particular immediate six-month period preceding the assessment, as well as the preceding six months. This correlation spanned across each PGY level, with EORd consistently having the strongest correlation in comparison to EOS or to EOR assessments completed by nurses (EORn). Over time, the strongest correlation of the final recommended proficiency level was found to be the immediate preceding proficiency level assigned by the CCC. In our CCC meetings, previous proficiency levels were available both during pre-review of the resident data, as well as during the discussion of current assignments. Given this finding, it may be prudent to withhold this information in future meetings to see whether or not the CCC members are biased by prior data.

In discussing the weak correlation between the final CCC-assigned proficiency levels and EOS assessments, Dehon et al. commented that their overestimation was likely related to a lack of “No” responses by faculty and re-calculated proficiency levels after including “N/A” as a “No” response,^4^ which allowed for a slightly increased differentiation across PGY level. At our program, we also noticed a paucity of “No” replies. This was thought to be related to faculty concern regarding the stigma associated with “No,” especially in that EOS assessments were suggested for use as a discussion point with the residents at the end of the shift. Therefore, we chose to modify our answer scale to non-dichotomous choices, allowing for a “Progressing” option, placed between a newly titled “Consistently Demonstrating” to replace “Yes” and “No,” which was replaced with an “Emerging” option. We chose “Emerging” as an attempt to remove the stigma associated with “No.” We allowed an “NA” option. Unlike Dehon, our rate of “No” or “Emerging” was unchanged (average rate 1.5%; range 0.6%–2.4%), with few faculty choosing this option regardless of the terminology used to describe it. We did, however, note a significant decrease in both the use of the “N/A” option, as well as in “Yes” or the newly titled “Consistently Demonstrating,” with an average usage of “Consistently Demonstrating” of 83.1% compared to 96.7% of “Yes” in the first year of the program. The “Progressing” option is responsible for the entirety of this difference. Despite this change, we noted no increase in the correlation of the EOS assessments with the final CCC proficiency level.

In evaluating EOR assessments, Kuo et al.^8^ described the use of a milestone-based evaluation system in a surgery residency program in which global assessments using selected subcompetencies were sent out at the end of resident rotations. The authors found that EOR assessments yielded an increased distribution of possible scores across PGY levels, with evaluators using a wider range of the scale, including the lower proficiency levels. This was compared to their traditional Likert scale assessments, in which the median composite PGY1 score was 3.63 on a 1–4 scale, in comparison to 1.88 (proficiency levels 1–4) in their new milestone-based system.

Similar to the findings of Kuo et al., our study demonstrated that our program’s EOR assessments, namely by doctors, reflected an increased distribution of scores, perhaps reflected in their higher correlations seen with our EOR and CCC proficiency levels. It is possible that the CCC may have found the EORd assessment to be more credible than other assessments and was biased towards considering these results more favorably. However, given the summative nature of both a global rating form and the milestones, it is perhaps not surprising that this is where we found the highest correlation.

### Assessment Tools Inter-Correlations

In addition to not correlating well with the CCC proficiency levels, we also found that the EOS assessments did not correlate well with their counterpart EOR assessments when compared by subcompetency. As our newly implemented evaluation program progressed, and perhaps due to continued re-education to nursing about the non-Likert scale of proficiency levels, EORn and EORd were more in line with each other. However, the EORn assessments continually yielded a more inflated overall score for residents than EORd. We found that nurses were highly resistant to assigning lower proficiency levels, even to PGY1 residents at the onset of the program. While our re-education did yield slightly lower overall scores on the whole, EORn assessments continued to rate residents quite higher on the proficiency scale. In general, the EORn assessment scores were felt to not be useful to CCC members in deciding on their final proficiency scores; however, all members felt the descriptive comments provided by nursing staff were invaluable in finding items for improvement and commendation. Given the Accreditation Council for Graduate Medical Education (ACGME) requirement for multiple assessors,^8^ it may be prudent to use feedback from nurses for more formative feedback, as opposed to the EORn assessments used in this initial version of our program.

### Correlations by Subcompetency

Our study found that whenever multiple data sources (EORd, EOS, and EORn) were used to assess an individual subcompetency, the correlation for the CCC proficiency levels across all of these subcompetencies was also highest with the EORd compared to the two other data sources.

EOS assessments had the highest correlation with final CCC proficiency levels in milestones from PC3 (Diagnostic Studies) and PC7 (Disposition), while the lowest correlations were seen in those from SBP1 (Patient Safety) and PROF2 (Accountability). There were no strong correlations for either of the Interpersonal and Communication subcompetencies (Patient Communication or Team Management), nor either of the Professionalism subcompetencies between EOS assessments and final CCC proficiency levels. We found this particular weak correlation surprising, given that direct observation should provide the best opportunity for accurate assessments of skills such as communication and professionalism. We suspect that the variety of a resident’s clinical encounters during any given shift may contribute to these data. Due to this finding, we advocate that EOS assessments be used cautiously as individual data points reflecting a “snapshot” of competence and not representative of a trainee’s global assessment, to ensure the data provided can capture multiple encounter opportunities.

## LIMITATIONS

We collected our data at a single site using two main assessment tools. While the CCC had an increased number of data points available for use, it is possible that the format used by our CCC is not generalizable to other institutions. In addition, the EOS is a paper tool, which is not ideal. However, we believe it is feasible to sustain use of the instrument as a paper tool if desired, as we have been using it now for over three years. Ideally, the tool would become an electronic assessment that would be completed in real time. We cannot infer how this would change the utility of the tool or its correlation to CCC levels.

In some instances, individual residents may have limited assessment data. Over the PGY1 year, our interns spend less than half of their year on ED rotations and some may have had minimal exposure during each individual six-month time period. Due to this variable pattern of resident schedules, as well as the small number of expected assessments over a single experience, we did not compare assessment data month to month, but rather over six-month periods. We felt this was not a significant limitation, given the data is being used for CCC discussions, which occur only every six months. Similarly, overall nursing data collected contributed to the smallest percentage of our individual assessment tools. However, we believe nursing assessments are an important component for trainee assessment, given the ACGME’s requirement for multisource assessments by multiple evaluators, including professional staff.

Lastly, as residents are allowed to select faculty for the EORd assessments, it is possible that this self-selection has skewed our data. We did, however, note that our most “critical” faculty were frequently chosen and believe residents selected a wide variety of assessors over time. Any faculty is able to trigger and complete an assessment at any time in the electronic system.

## CONCLUSION

In our single center study of assessing EM residents’ milestone proficiency, the end-of-rotation (EORd) assessments completed by supervising physicians (attendings and senior residents) are the most highly correlated with the final CCC proficiency level designation, while end-of-shift (EOS) assessments and end-of- rotation assessments by nurses (EORn) did not correlate well with final CCC proficiency levels. Every level of proficiency the CCC assigned appears to be highly correlated with the designated level in the immediate six-month period, perhaps implying CCC members are biased by previous level assignments. Based on our study, we advocate that EOS assessments be used cautiously as individual data points reflecting a “snapshot” of competence and not representative of a trainee’s global performance. Further studies are needed to determine the utility of the EOS for CCC use, and the effect of blinding of prior CCC-assigned proficiency levels on current proficiency level designation.

## Figures and Tables

**Table 1:** End-of-rotation (EOR) and end-of-shift (EOS) assessment completion rates per resident/per year.

	PGY 1	PGY2	PGY 3	PGY 4
2014 EORd	6–10, median=9	12–18, median=16	14–19, median=18	14–22, median=21
2015 EORd	4–7, median=6	11–17, median=13	14–17, median=16	18–23, median=20
2014 EORn	4–8, median=6	8–12, median=10	10–14, median=12	12–18, median=18
2015 EORn	3–6, median=5	6–11, median=9	9–13, median=11	7–11, median=10
2014 EOS	28–56, median=40	15–76, median=47	18–87, median=34	17–59, median=36
2015 EOS	8–57, median=25	13–53, median=36	12–55, median=38	10–38, median=25

*EOS*, end of shift; *EORd*, end of rotation (doctor); *EORn*, end of rotation (nurse); *PGY*, post graduate year.

Completion rates are per resident/per year listed by min-max, median.

**Table 2:** Correlation table of end-of-rotation assessments by nurses and doctors vs. end-of-shift assessments for 24 months.

	EORd.D2013		EORd.M2014		EORd.D2014		EORd.M2015	
			
EOS.D2013	0.49	p<0.0005*	0.56	p<0.0005*	0.65	p<0.0005*	0.65	p<0.0005*
EOS.M2014	0.38	p<0.0005*	0.47	p<0.0005*	0.63	p<0.0005*	0.62	p<0.0005*
EOS.D2014	0.12	p=0.02	0.14	p=0.003	0.61	p<0.0005*	0.58	p<0.0005*
EOS.M2015	0.01	p=0.93	0.07	p=0.21	0.34	p<0.0005*	0.45	p<0.0005*
	EORn.D2013		EORn.M2014		EORn.D2014		EORn.M2015	
				
EOS.D2013	0.37	p<0.0005*	0.52	p<0.0005*	0.48	p<0.0005*	0.39	p<0.0005*
EOS.M2014	0.29	p<0.0005*	0.36	p<0.0005*	0.46	p<0.0005*	0.3	p<0.001
EOS.D2014	0.1	p=0.06	0.29	p=0.001	0.54	p<0.0005*	0.27	p<0.000
EOS.M2015	−0.17	p=0.18	0.20	p=0.08	0.34	P=0.001	0.39	p<0.0005*

*EOS*, end of shift; *EORd*, end of rotation (doctor); *EORn*, end of rotation (nurse); *D*, December; *M*, May.

Using Bonferonni correction, p values less than 0.0005 are considered statistically significant and have been designated with an asterisk (*)

**Table 3:** Correlation table of EORn versus EORd for 24 months.

	EORn.D2013		EORn.M2014		EORn.D2014		EORn.M2015	
			
EOS.D2013	0.57	p<0.0005*	0.31	p<0.0005*	0.52	P=0.0008	0.31	p=0.48
EOS.M2014	0.48	p<0.0005*	0.51	p<0.0005*	0.59	p<0.0005*	0.37	p=0.64
EOS.D2014	0.61	p<0.0005*	0.51	p<0.0005*	0.7	p<0.0005*	0.67	p<0.0005*
EOS.M2015	0.4	p=0.48	0.59	p<0.0005*	0.7	P=0.001	0.65	p<0.0005*

*EOS*, end of shift; *EORd*, end of rotation (doctor); *EORn*, end of rotation (nurse); *D*, December; *M*, May.

Using Bonferonni correction, p values less than 0.0005 are considered statistically significant and have been designated with an asterisk (*)

**Table 4:** Correlation table of EOS and EOR versus final CCC assigned level of proficiency.

	EOS.D2013	EOS.M2014	EOS.D2014	EOS.M2015
CCC.D2013	0.49			
CCC.M2014	0.51	0.53		
CCC.D2014	0.57	0.62	0.6	
CCC.M2015	0.53	0.59	0.58	0.49
	EORd.D2013	EORd.M2014	EORd.D2014	EORd.M2015
				
CCC.D2013	0.71			
CCC.M2014	0.7	0.85		
CCC.D2014	0.54	0.77	0.84	
CCC.M2015	0.46	0.71	0.81	0.84
	EORn.D2013	EORn.M2014	EORn.D2014	EORn.M2015
				
CCC.D2013	0.53			
CCC.M2014	0.5	0.56		
CCC.D2014	0.47	0.55	0.73	
CCC.M2015	0.4	0.54	0.68	0.64

*CCC,* clinical competency committee; *EOS*, end of shift; *EORd*, end of rotation (doctor); *EORn*, end of rotation (nurse); *D*, December; *M*, May.

Using Bonferonni correction, p values less than 0.0005 are considered statistically significant.

Blank areas represent data not available for correlated CCC.

**Table 5:** Correlation table of final CCC proficiency levels over time.

	CCC.D2013	CCC.M2014	CCC.D2014	CCC.M2015
CCC.D2013	1.00	0.94	0.78	0.71
CCC.M2014	0.94	1.00	0.83	0.76
CCC.D2014	0.78	0.83	1.00	0.92
CCC.M2015	0.71	0.76	0.92	1.00

*CCC,* clinical competency committee; *D*, December; *M*, May.

Using Bonferonni correction, p values less than 0.0005 are considered statistically significant.

**Table 6:** Correlated CCC proficiency levels across PGY levels and subcompetencies.

	CCC_EORd	CCC_EOS	CCC_EORn
PGY1	0.815	0.56	0.59
PGY2	0.83	0.49	0.57
PGY3	0.85	0.49	0.515
PGY4	0.74	0.52	0.71
ICS1	0.8	0.59	0.66
ICS2	0.81	0.57	0.71
MK			
PBLI			
PC1	0.85	0.64	
PC10	0.70		
PC11	0.72		
PC12			
PC13	0.83		
PC14	0.83		
PC2	0.83	0.51	
PC3	0.86	0.72	
ICS1	0.8	0.59	0.66
ICS2	0.81	0.57	0.71
MK			
PBLI			
PC1	0.85	0.64	
PC10	0.70		
PC11	0.72		
PC12			
PC13	0.83		
PC14	0.83		
PC2	0.83	0.51	
PC3	0.86	0.72	

*CCC,* clinical competency committee; *EOS*, end of shift; *EORd*, end of rotation (doctor); *EORn*, end of rotation (nurse); *ICS,* interpersonal and communication skills; *MK,* medical knowledge; *PBLI,* practice-based learning and improvement; *PC,* patient care; *PROF,* professionalism; *SBP,* system-based practice.

Using Bonferonni correction, p values less than 0.0005 are considered statistically significant.

Blank areas represent areas where the subcompetencies were not evaluated by the data source.

Average correlations across 24 months.

P-values were not calculated.
